# MSEI-ENet: A Multi-Scale EEG-Inception Integrated Encoder Network for Motor Imagery EEG Decoding

**DOI:** 10.3390/brainsci15020129

**Published:** 2025-01-28

**Authors:** Pengcheng Wu, Keling Fei, Baohong Chen, Lizheng Pan

**Affiliations:** School of Mechanical Engineering and Rail Transit, Changzhou University, Changzhou 213164, China; s22050802001@smail.cczu.edu.cn (P.W.); s22050858003@smail.cczu.edu.cn (B.C.); panlz@cczu.edu.cn (L.P.)

**Keywords:** multi-scale structure, inception, transformer, motor imagery, brain–computer interface

## Abstract

Background: Due to complex signal characteristics and distinct individual differences, the decoding of a motor imagery electroencephalogram (MI-EEG) is limited by the unsatisfactory performance of suboptimal traditional models. Methods: A subject-independent model named MSEI-ENet is proposed for multiple-task MI-EEG decoding. It employs a specially designed multi-scale structure EEG-inception module (MSEI) for comprehensive feature learning. The encoder module further helps to detect discriminative information by its multi-head self-attention layer with a larger receptive field, which enhances feature representation and improves recognition efficacy. Results: The experimental results on Competition IV dataset 2a showed that our proposed model yielded an overall accuracy of 94.30%, MF1 score of 94.31%, and Kappa of 0.92. Conclusions: A performance comparison with state-of-the-art methods demonstrated the effectiveness and generalizability of the proposed model on challenging multi-task MI-EEG decoding.

## 1. Introduction

The motor imagery brain–computer interface (MI-BCI), as an innovative paradigm, has received significant attention for its potential usage in the medical rehabilitation field and critical situations. Without the actual performance of a movement, a MI-BCI can decode electroencephalography (EEG) signals of a subject’s brain activity and convert them to relevant commands; thus, it can assist in to controlling external devices, such as wheelchair and prosthetics, and can further promote motor function recovery in patients post-stroke [1]. MI-BCI decoding involves deciphering useful and reliable information from mixed and sometimes weak signals from multiple electrodes. MI-BCI decoding constitutes a crucial component in the entire system and influences the recognition efficacy of MI tasks greatly. 

Because of its merits of high temporal resolution, non-invasiveness, and low cost, scalp EEG is the most common method for the acquisition of brain neural dynamics. However, these weak signals have distinct non-stationary and nonlinear characteristics, and spatial coupling intricates the effective representation of specific motor intention. Individual heterogeneity also poses a challenge for MI-EEG decoding [2,3,4].

MI can activate specific cortical areas similar to actually performing relevant movement, and it induces event-related desynchronization and synchronization (ERD/ERS) in EEG patterns [5]. The ERD/ERS pattern represents a decrease or increase in the spectrum amplitude in a certain frequency band [6]. This can be used as an effective indicator that facilitates feature representation for MI-EEG decoding. Common spatial pattern (CSP) has been recognized as an effective algorithm to improve ERD/ERS detection [7]. Since its performance is dependent on a subject-specific frequency band, variants of CSP have been proposed [8,9,10], and discriminative information in the spectral domain could be obtained by a specific configuration of filter banks [11,12]. To reduce the dimension of features extracted by the CSP-based method, selecting appropriate features that are most relevant for specific motor imagery tasks is required. Zhang et al. [13] proposed CSP with a non-convex log function for feature selection. Jin et al. [14] used an improved CSP objective function to discover features with larger inter-class distances. These methods achieved not only the identification of discriminative spatial information but also spectral information, whereas the adaptability of these methods is constrained as the data volume and task complexity increase. 

The outstanding feature learning ability of convolutional neural networks (CNNs) attracts widespread attention in MI-EEG decoding. As a compact CNN, EEGNet can capture temporal and spatial domain information of EEG signals by using different convolution layers [15], and various models for MI task recognition have been developed using the EEGNet framework. Zhang et al. [16] built an inception-based neural network that could extract features parallelly. To reduce the complexity of network structure, Riyad et al. [17] utilized Mobile Inception EEGNet to learn rich features with a reasonable number of parameters. Wang et al. [18] proposed a method to enhance the ability of the network by embedding graph convolution. Ingolfsson et al. [19] added a temporal convolutional network (TCN) to EEGNet to more efficiently process the time series. Salami et al. [20] improved EEG_TCNet by using inception modules and dilated causal convolution. These models achieved encouraging results for subject-dependent MI-EEG decoding by taking advantage of different convolution layers. Some studies utilize multi-branch CNNs to decode MI-EEG signals. Jia et al. presented a multi-branch multi-scale CNN framework (MMCNN) to extract features from multiple sizes of the convolution kernel [21]. Tang et al. proposed a multi-scale hybrid network to extract spatiotemporal features efficiently by adopting a method of feature enhancement and achieved improved performance on different datasets [22]. Additionally, certain studies have focused on attention mechanisms for their ability to exploit interconnections between features. By adopting a multi-head attention mechanism of Transformer combined with CNN, Song et al. [23] proposed a Conformer to address the drawback that the CNN framework could only extract local features and was unable to capture the long-term dependencies in EEG decoding. To further overcome the issue that the Transformer fails to capture the temporal dynamics within EEG signals, Ding et al. [24] developed a Deformer that introduced hierarchical Transformer blocks to capture the temporal patterns of EEG signals and adopted a dense information purification module to enhance the model performance.

From the perspective of experimental paradigms of MI-EEG decoding, the commonly adopted methods can be classified into two major categories: subject-dependent and subject-independent. The former trains a model separately for each subject and can capture the unique features of a specific subject. However, this incurs high computational costs since it requires training for a single individual. By comparison, subject-independent models are trained on the data from multiple individuals with better generalization, which can adapt to different individuals without additional adjustment [25]. There are two primary ways to evaluate the subject-independent model. One is to utilize data from all subjects to form a monolithic dataset with a portion for evaluation [26], named global cross-validation (global-CV). Under the global-CV scenario, Fan et al. [27] proposed a bilinear neural network and achieved good results. Zhang et al. [28] presented a deep CNN for different kinds of binary classification MI experiments. They used ten-fold cross-validation on four datasets to evaluate the robustness of the model. The other one is called leave-one-subject-out cross-validation (LOSO-CV). Kwon et al. [29] applied a deep CNN for left- and right-hand MI classification. Luo et al. [25] utilized a shallow mirror Transformer (SMTransformer) to achieve good performance on binary classification MI tasks with improved generalization. Such a subject-independent model can learn features comprehensively and attain research focus with the justification that it offers flexibility for MI-BCI applications without requiring a user-specific calibration process [11]. However, the subject-independent method does not perform as well as the subject-dependent method. Particularly, when multi-task MI-EEG decoding involves lower limbs, it becomes more challenging than upper limb binary classification tasks because of the difficulty in learning distinguishable features.

This paper proposes a subject-independent model multi-scale EEG-inception integrated encoder Network (MSEI-ENet) for multi-task MI-EEG decoding. Firstly, the multi-scale EEG-inception (MSEI) module is employed to extract the spectral and spatial features of an MI-EEG. Furthermore, its multiple convolution blocks with different scales are used to obtain diversified frequency information from the raw EEG signals. Secondly, the encoder module utilizes the multi-head self-attention mechanism to identify interrelationships among the input features and assigns different weights to the features based on their importance. The recognized BCI Competition IV dataset 2a and the Physionet dataset are used to validate the effectiveness of the proposed model. 

## 2. Materials and Methods

### 2.1. Dataset and Preprocessing

This paper utilizes BCI Competition IV dataset 2a (BCIIV 2a) and Physionet datasets for verification of the proposed model [30].

#### 2.1.1. BCI Competition IV Dataset 2a

**Figure 1:**
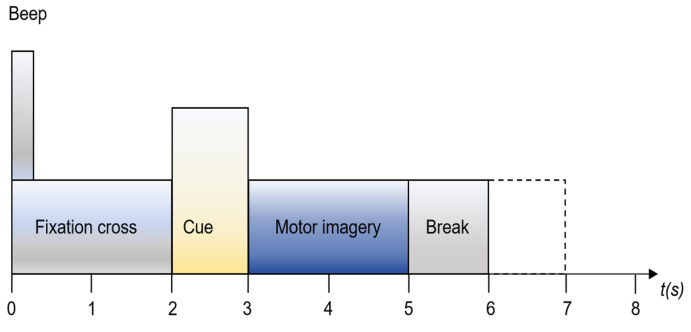
Motor imagery paradigm for BCIIV2a.

The datasets for the MI-EEG included nine individuals and four motor imagery tasks (left hand, right hand, both feet, and tongue). Data were collected from two sessions on different days using a sampling frequency of 250 Hz and 22 electrodes [31]. Each session comprised 288 EEG trials, with 72 trials per task. The participants were instructed to perform the motor imagery task until the fixation cross disappeared from the screen, as illustrated in Figure 1. For our experiments, 4s data corresponding to the actual duration of the motor imagery task was used. Notably, we only selected three channels (C3, Cz, and C4) since these channels are closely related to the motor cortex and a reduced number of channels helps accelerate data processing. 

#### 2.1.2. Physionet Dataset

The datasets for the MI-EEG included 109 individuals and four motor imagery tasks (left hand, right hand, both hands, and both feet) [32]. There were 64 electrodes based on the international 10-10 system. Each subject performed 84 trials (3 sessions × 7 trials × 4 tasks). The sampling rate was 160 Hz, and the EEG signals were recorded with a duration of 4 s (640 sample points for each trial). For our experiments, 3 channels (C3, Cz, and C4) and 4s data were used. Due to memory constraints, 20 subjects were randomly selected from the 109 individuals. 

#### 2.1.3. Preprocessing

Since MI-EEG experimental datasets are typically small, directly using them in deep learning models may cause overfitting. Data augmentation was performed on the raw EEG data to better fit the proposed model. The data augmentation included three main steps: (1) interpolation, (2) sliding window processing, and (3) Gaussian data augmentation. Given the raw data *X^N^^×^^C^^×^^T^*, where *N* indicates the number of trials, *C* indicates the number of channels, and *T* is the sample number, quadratic spline interpolation was employed. The entire process can be expressed by Equations (1) and (2):

(1) $$X^{N\times C\times U}\;=SI(X^{N\times C\times T}),\;X^{5N\times C\times L}\;=SW(X^{N\times C\times U},w,s)$$

(2) $$X_{G}=X+G(\mu ,\sigma ),\;X^{10N\times C\times L}=\{X^{5N\times C\times L},X_{G}^{5N\times C\times L}\}$$

where *SI* denotes quadratic spline interpolation, *SW* represents the operation of sliding window, *U* indicates the sample number after interpolation, and *L* is the sample number after sliding window. Specifically, the window length *w* was set to 1000 and the stride *s* was set to 10. *G* denotes a random Gaussian variable with mean *μ* = 0 and standard deviation *σ* = 0.005. Consequently, the augmented datasets were formed with a combination of Gaussian data *X_G_* and raw data *X*. For global-CV and LOSO-CV experiments, data augmentation was only performed on the training set to avoid data leakage. The detailed configuration of the BCIIV2a and Physionet datasets are shown in Table 1.

### 2.2. MSEI-ENet

**Figure 2:**
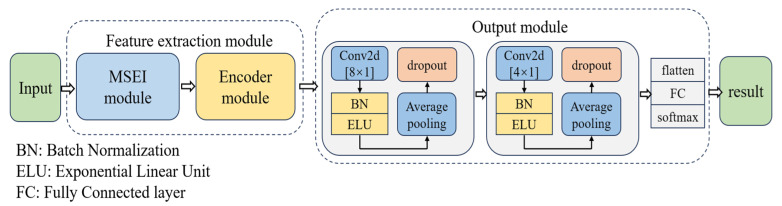
Overall framework of the proposed model for MI-EEG classification.

The overall framework of our proposed model is illustrated in Figure 2. The feature extraction module consists of two main components: (1) the MSEI module and (2) the encoder module. The former serves as the original feature extractor, and the extracted features are used as input to the encoder module, which is from the tailored Transformer. Finally, the output module is used to reduce the feature dimensionality and then output the final classification. 

#### 2.2.1. Multi-Scale EEG-Inception Module 

**Figure 3:**
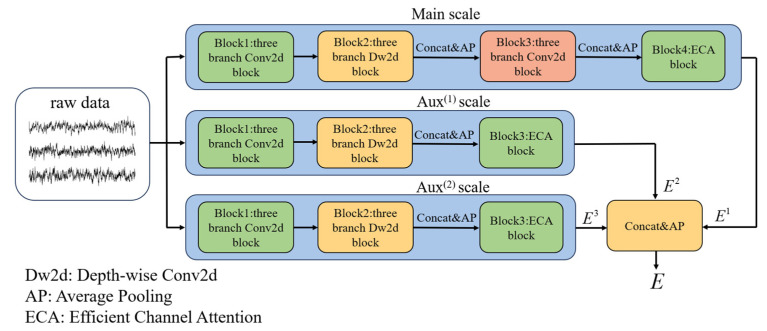
Overall architecture of the multi-scale EEG-inception module.

Unlike traditional CNNs, the inception network can capture rich information by performing multiple convolution and pooling operations on input images in parallel, which enhances image representation [33]. The EEG-inception network inherits these qualities, and the multi-branch structure enables the extraction of features from both spectral and spatial domains [34]. Therefore, we adopted it as the backbone of our MSEI module for feature extraction in MI-EEG decoding. The overall architecture of the MSEI module is illustrated in Figure 3. This module has a multi-scale structure, which consists of the Main scale and two other auxiliary scales, called Aux^(1)^ and Aux^(2)^. Specifically, given a training set {*X*, *Y*}, *X* is a tensor ∈R*^L×C^* (*L* is the sample number that is set to 1000 for BCIIV2a and 640 for Physionet), which denotes the data of the selected EEG signals, and *Y* denotes the label of training data. When *X* is input to the network, it will be fed into the three scales parallelly to achieve feature extraction. 

**Figure 4:**
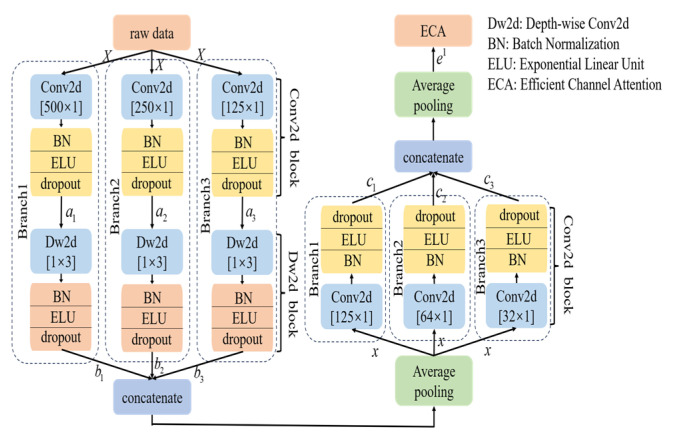
Schematic illustration of the Main scale.

The specific structures of the Main scale are shown in Figure 4. Firstly, raw data *X* are fed into a three-branch Conv2d block with different convolution kernel sizes. In these Conv2d layers, padding is set to “same” to ensure that the dimension of output is consistent with that of its input, facilitating the concatenation of features. Subsequently, batch normalization (BN), which follows the three-branch Conv2d block, is used to normalize the distribution of the feature maps. The exponential linear unit (ELU) is adopted as an activation function to introduce nonlinearity and a dropout layer to avoid overfitting. This process can be represented as:

(3) $$a_{i}=F\;[Conv2d(X)]$$

where *F*[*Conv*2*d*(*X*)] represents the operations of the Conv2d block and *a_i_* denotes the output features of the Conv2d block; *I* ∈ {1, 2, 3} indicates the three branches of the Main scale. Since MI induces event-related desynchronization and synchronization (ERD/ERS) in EEG patterns [35], the MSEI module attempts to learn such spectral information from the raw signals at different scales. 

Secondly, the three-branch depth-wise Conv2d (Dw2d) block is used to extract spatial features among the channels. Considering the data are collected from multi-channel electrodes placed on the scalp, the effect of volume conduction may cause the spatial mixture of MI-EEG signals [4,36], and the adoption of Dw2d could make the model learn the spatial information of the MI-EEG. Since three-channel EEG signals are used in this study, the kernel size of Dw2d is set to [1 × 3].

Subsequently, a concatenation layer is used to merge the extracted features *b_i_* into *x*, and then an average pooling layer (Ap2d) is used to reduce the dimension of features. The concatenated features *x* can be expressed as:

(4) $$b_{i}=F[Dw2d(a_{i})],\;x=Ap2d\;[concat(b_{1},b_{2},b_{3})]$$

where *F*[*Dw*2*d*(*a_i_*)] denotes operations of the Dw2d block.

Furthermore, a three-branch Conv2d block is put after Ap2d to explore deeper features of EEG signals. We concatenate the output *c_i_* ($c_{i}=F[Conv2d(x)]$) of the Conv2d block and then use Ap2d to reduce the dimension of features; the output feature *e*^1^ is:

(5) $$e^{1}=Ap2d[concat(c_{1},c_{2},c_{3})]$$

**Figure 5:**
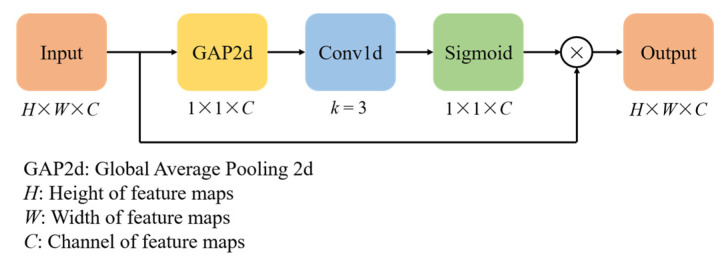
Structure of ECA.

Finally, Efficient Channel Attention (ECA) is used in the Main scale. As a channel attention mechanism, ECA can learn the importance of each channel in the network and help focus on the critical feature channels [37]; its architecture is shown in Figure 5. For the ECA, the kernel size of Conv1d is adaptively determined according to the number of channels in the input feature maps. The kernel size is $k=|\frac{{log}_{2}\left(C\right)}{\gamma }+\frac{b}{\gamma }|$, where *C* denotes the number of channels and the constants are *γ* = 2, *b* = 1. The specific parameters of the Main scale are presented in Table 2. The final output feature *E*^1^ is expressed as:

(6) $$E^{1}=ECA(e^{1})$$

The Aux^(1)^ and Aux^(2)^ scales are used to complement the Main scale for comprehensive feature representation. Unlike the Main scale, these two auxiliary scales have only one three-branch Conv2d block, thereby reducing the complexity of the network without sacrificing the overall accuracy. The specific parameters of the Aux^(1)^ scale are presented in Table 3. The kernel sizes of the convolution layers of the Aux^(2)^ scale are [32 × 1], [16 × 1], and [8 × 1], while the other parameters are consistent with those of the Aux^(1)^ scale.

Finally, the output feature *E* of the multi-scale EEG-inception module is calculated as:

(7) $$E=Ap2d[concat(E^{1},E^{2},E^{3})]$$

where *E*^1^, *E*^2^, and *E*^3^ represent the features extracted from the Main, Aux^(1)^, and Aux^(2)^ scale, respectively.

#### 2.2.2. Encoder Module

When multi-task MI-EEG decoding involves lower limbs such as both feet, because the motor cortex of a lower limb MI task might be located in a deep brain area, it becomes challenging to extract distinguishable features [38,39]. The success of self-attention and Transformer in calculating the relevance among features with a large receptive field inspired us [40,41,42,43]. We only adopted the encoder module to reduce the complexity of the model, using its multi-head attention mechanism to detect discriminative information of the features that are extracted from the MSEI module. 

**Figure 6:**
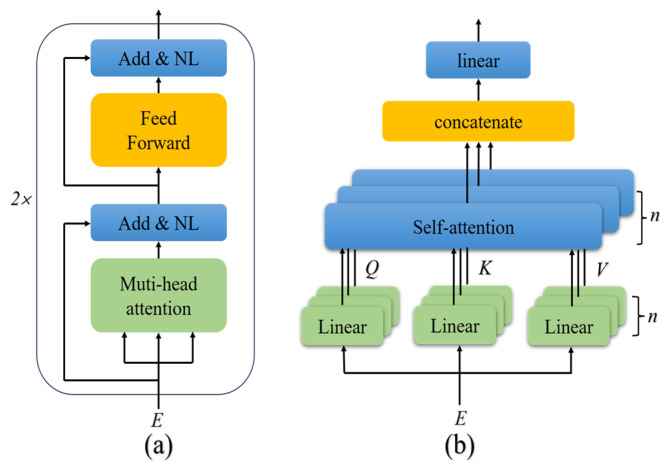
Schematic illustration of the encoder module: (**a**) its layer and (**b**) multi-head attention mechanism.

The encoder module consists of two identical layers, each comprising a multi-head attention sub-layer and a feed-forward sub-layer, as shown in Figure 6a. A residual structure is adopted in each sub-layer to avoid vanishing gradients and weight matrix degradation. Subsequently, layer normalization was employed for gradient stabilization during optimization, which can overcome the challenge of inconsistent input distribution.

(1) Multi-head attention sub-layer

The multi-head attention sub-layer allows the model to focus information from different representation subspaces at different positions [44]. *E* is the input of the multi-head attention sub-layer. Query (*Q*), key (*K*), and value (*V*) represent matrices obtained by linearly projecting the input feature *E*. By calculating the similarity between *Q* and *K*, the weight of *V* is obtained. *Q*, *K*, and *V* can be calculated as:

(8) $$Q=EW^{Q},\;K=EW^{K},\;V=EW^{V}$$

where *W^Q^*, *W^K^*, and *W^V^* represent the projection matrices of *Q*, *K*, and *V*, respectively. The self-attention equation can be calculated as:

(9) $$Attention(Q,K,\;V)=SoftMax(\frac{QK^{T}}{\sqrt{d_{k}}})V$$

where *d_k_* represents the dimension of matrix *K*. The value obtained after the dot product of *Q* and *K* must be divided by a scaling factor $\sqrt{d_{k}}$, which could avoid the vanishing gradient caused by the notably large inner product. 

Since MI-EEG signals are often complex and varied, the adoption of multi-head attention can increase the diversity of features and improve the generalization of the network, in comparison with the limited information captured by single self-attention. Multi-head attention improves the self-attention mechanism by dividing the input features into *n* heads, where self-attention is performed in parallel on each head, as shown in Figure 6b.

(2) Feed-forward sub-layer

A fully connected feed-forward layer is added after the multi-head attention layer to enable the model to learn more complex and refined feature representations. This layer consists of two linear transformations represented by:

(10) $$FFN(z)=\max(0,zW_{1}+b_{1})W_{2}+b_{2}$$

where max represents the rectified linear unit (ReLU) activation, which introduces nonlinearity to enhance the representation ability of the features. The input of the first fully connected layer is defined as *z*. Variables *W*_1_ and *b*_1_ are the weights and biases of the first fully connected layer; *W*_2_ and *b*_2_ are those of the second fully connected layer. Through the first linear transformation, the features are mapped to higher dimensions, which makes them finer to improve the discriminative ability of the network. The second linear transformation reduces the dimensions of the features.

#### 2.2.3. Output Module

The output module mainly comprises two convolution layers and a classification layer. The convolution kernel sizes of the final two convolution layers are [8 × 1] and [4 × 1], respectively. In the classification layer, the output is flattened and then fed into the fully connected layer to predict the labels. The architectural details of the principal layers of MSEI-ENet are shown in Table 4.

### 2.3. Evaluation Metrics

We adopted the confusion matrix, precision (*PR*), recall (*RE*), and *F*1-score (*F*1) as evaluation metrics to conduct a comprehensive evaluation of our model. These metrics are calculated as:

(11) $$PR=\frac{TP}{\left(TP+FP\right)},\;RE=\frac{TP}{\left(TP+FN\right)}\;,\;F1=\frac{2*PR*RE}{\left(PR+RE\right)}$$

where *PR* indicates the proportion of true positive predictions among all positive results and *RE* refers to the proportion of true positive predictions among all actual positive cases. The *F*1-score combines the output results of *PR* and *RE* to provide a balanced measure for the performance of the model.

Additionally, the overall accuracy (*ACC*), macro *F*1-averaging (*MF1*), and Kappa values are also calculated to evaluate the performance:

(12) $$ACC=\frac{TP+TN}{\left(TP+TN+FP+FN\right)},\;MF1=\frac{1}{M}\sum_{i}F1_{i},\;kappa=\frac{P_{0}-P_{e}}{1-P_{e}}$$

where *M* is the number of classes and *P*_0_ and *P_e_* are actual and chance agreement, respectively.

## 3. Experiments and Results

### 3.1. Implementation Details

The task implementation was based on the TensorFlow framework, using Windows 10 and the Python 3.7.0 platform, with an NVIDIA GTX 1660 Ti graphics card. The Adam optimizer and cross-entropy loss function were adopted, with a learning rate of 0.0001 for the binary MI task and 0.0005 for the multi-task MI-EEG decoding. The dropout rate was set to 0.3 for the encoder module and 0.25 for the remaining modules. The head number *h* was set to 5 for the multi-head attention mechanism. The training epoch was set to 100, and early stopping was applied in the training stage to avoid overfitting. The batch size was set to 128 for the binary MI task and 256 for the multi-task experiments.

Additionally, we conducted global-CV and LOSO-CV experiments to evaluate our proposed model [26]. For global-CV experiments, 5-fold cross-validation was employed, in which the data from all subjects were split into five folds, one for the test set and the others for the training and validation sets. The binary (left/right-hand) MI-EEG decoding experiments were conducted first, and then multi-task MI-EEG decoding experiments were conducted on the BCIIV2a dataset. Second, to validate the model’s performance on other datasets, the multi-task MI-EEG decoding experiments were conducted on Physionet. The performance metrics obtained from related experiments were averaged to obtain the overall evaluation results. For LOSO-CV experiments, the data from one subject were used for the test set, and the data from the remaining subjects were used for the training and validation sets. The multi-task MI-EEG decoding experiments were conducted on BCIIV2a.

### 3.2. Results

#### 3.2.1. Experimental Results of MI-EEG Decoding

Since left/right-hand MI are classic tasks in motor imagery BCI, the left/right-hand MI-EEG decoding experiment was conducted first. Table 5 lists the confusion matrix of the proposed MSEI-ENet applied on left- and right-hand MI-EEG recognition on BCIIV 2a. It can be observed from Table 5 that the metrics of *PR*, *RE*, and *F*1 for left/right-hand MI tasks all achieve or exceed 98%, and the performance of our model on left- and right-hand MI tasks is comparable, with *F*1-scores of 98.26% and 98.27%. This shows that our proposed model performs well in left- and right-hand MI decoding.

Additionally, we also conducted a multi-task MI-EEG decoding experiment involving left-hand, right-hand, both-feet, and tongue motor imagery. The implementation details are consistent with the left/right-hand experiment. Table 6 lists the confusion matrix of the proposed MSEI-ENet applied on BCIIV 2a for multi-task MI-EEG decoding. It is observed that the *RE* values of the left- and right-hand MI tasks achieve 93.44% and 95.37%, respectively, which are lower than the results obtained for the left/right-hand classification tasks (Table 5). Notably, the *F*1-scores of multi-task MI are similar, reaching approximately 94%, which is slightly lower compared to the left/right-hand MI tasks. It is noteworthy that the *RE* of the both-feet MI task is the highest, reaching 95.75%. Although the *RE* of the tongue MI task is the lowest at 92.66%, its precision (*PR*) is the highest at 96.00%. It can be seen that MSEI-ENet also exhibits excellent performance in multi-task MI-EEG decoding, particularly in the challenging lower-limb MI task. Moreover, misclassification is more severe for four MI tasks compared to the left/right-hand MI tasks. This stems from the fact that the motor cortex of the lower-limb MI task might be located in a deep brain area, which is challenging.

To further validate the generalization of the proposed model, the multi-task MI-EEG decoding experiment was then conducted on the Physionet dataset. Table 7 shows the confusion matrix and the related performance indices. The *PR*, *RE*, and *F*1-score of the both-feet MI task achieve 100%, 98.81%, and 99.40%, respectively, which indicates that MSEI-ENet performs well on both-feet MI tasks. Except for the *PR* of 94.74 for the both-hands MI task, all other metrics of left/right hand and both hands are lower than 90%. It can be seen from the confusion matrix that the proportion of misclassifications between left- and right-hand MI tasks is relatively high, and labels of the both-hands task are misclassified to those of the left or right hand. The reason for this phenomenon may be that when the both-hands MI task is involved, it makes the recognition of upper-limb MI tasks complex.

**Figure 7:**
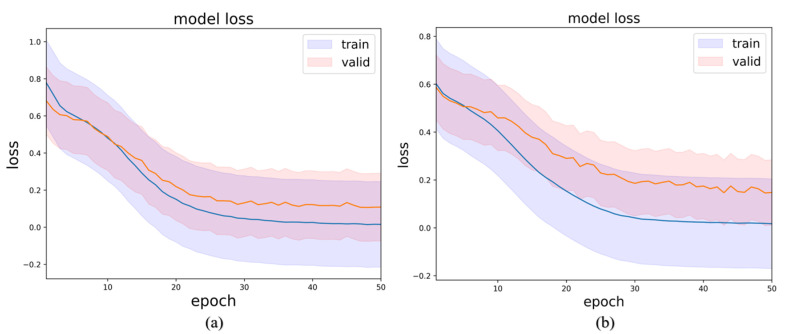
Training and validation loss trends during training epochs of the proposed model on BCIIV 2a for (**a**) left/right-hand binary classification and (**b**) multiple classification.

In order to evaluate the fitting performance of the MSEI-ENet model, the average loss within 50 epochs during the training course was calculated. For left/right-hand binary classification and multiple classification tasks, the training and validation loss on BCIIV2a are shown in Figure 7. The shaded regions of the training and validation loss curves are obtained by calculating the standard deviations of the loss, which exhibit the stability of the model. Notably, the loss curve levels off after the 30th epoch, and the validation loss value is below 0.2, which indicates that the proposed model rapidly converges to a stable value.

**Figure 8:**
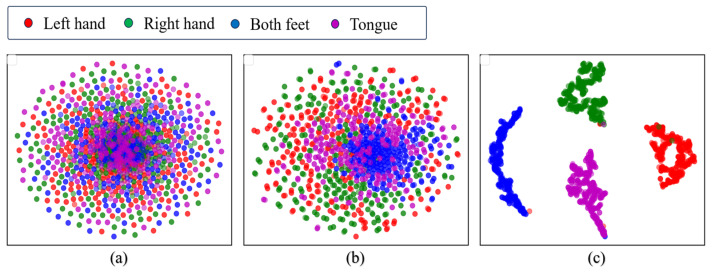
Visualization with *t*-SNE on the test set of the BCIIV2a dataset: (**a**) raw data; (**b**) features extracted by the MSEI module; (**c**) features extracted by the encoder module.

To provide an intuitive understanding of the features learned by our proposed model, we visualized the extracted features from the relevant modules using *t*-distributed stochastic neighbour embedding (*t*-SNE) [45]. This technique reduces the dimensionality of high-dimensional features. Figure 8 displays the *t*-SNE visualization of the raw MI-EEG data and the visualization of the features extracted by the MSEI module, as well as those extracted by the encoder module. It can be observed from Figure 8a that the raw MI-EEG data are difficult to cluster. As shown in Figure 8b, there is still some mixing among the features extracted by the MSEI module. In contrast, Figure 8c demonstrates that features from each class can be clearly distinguished. This indicates that when the model uses only a single feature extraction module, it has limited learning capability, whereas the combination of MSEI with the encoder module further enhances the feature representation, thereby improving the overall discriminative ability of the model.

#### 3.2.2. Ablation Experiment

(1) Comparison of MSEI-ENet modules

**Figure 9:**
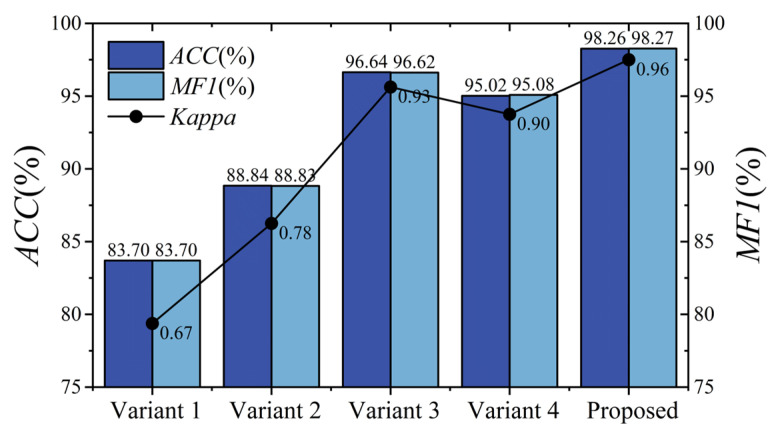
Performance comparison among Variant 1, Variant 2, and Variant 3 for binary classification on BCIIV 2a.

**Figure 10:**
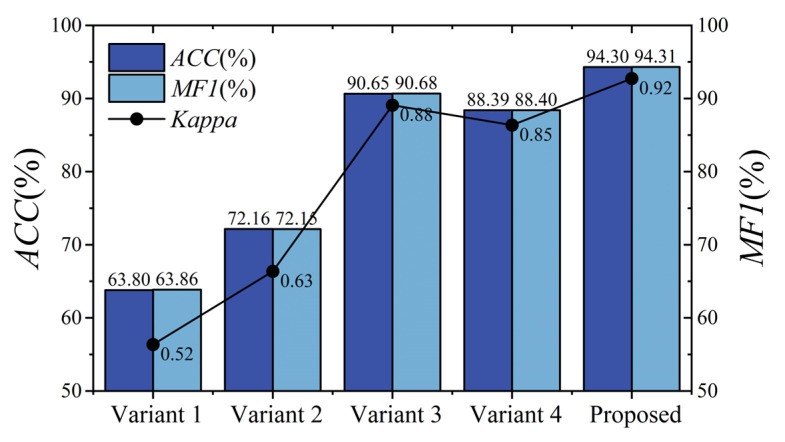
Performance comparison among Variant 1, Variant 2, and Variant 3 for multiple classification on BCIIV 2a.

**Figure 11:**
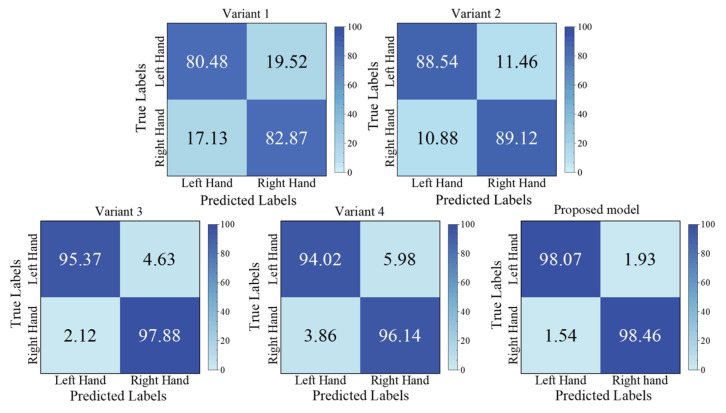
Confusion matrices corresponding to the comparison results for binary classification.

**Figure 12:**
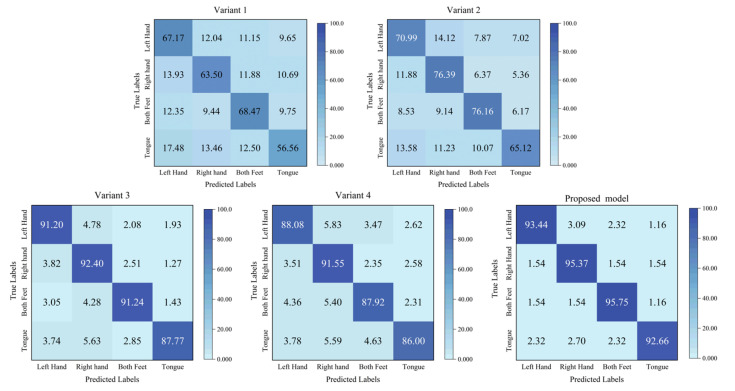
Confusion matrices corresponding to the comparison results for multiple classification.

The proposed model contains two crucial modules, as described in Section 2.2. To validate their effectiveness, we conducted ablation experiments on BCIIV 2a. Two variant models were designed. For variant 1, the Aux^(1)^ and Aux^(2)^ scales of MSEI and the encoder were removed; for variant 2, only the encoder was removed. The details of the experimental setup are listed in Table 8. The results of the ablation experiment on BCIIV 2a of the binary and multiple classification tasks are illustrated in Figure 9 and Figure 10. Furthermore, the corresponding confusion matrices of the variant models are shown in Figure 11 and Figure 12.

As shown in Figure 9 and Figure 10, for the left/right-hand MI task, the proposed model achieves an overall accuracy of 98.26%, an MF1 value of 98.27%, and a kappa value of 0.96. For the multiple classification task, the proposed model attains an overall accuracy of 94.30%, an MF1 value of 94.31%, and a kappa value of 0.92. Furthermore, Variant 2 shows a slight improvement over Variant 1 in both binary left/right-hand and multi-task MI-EEG decoding because the multi-scale structure captures comprehensive information compared to a single structure. Notably, for the left/right-hand MI task, the accuracies of Variant 1 and Variant 2 are lower than that of the proposed model by 14.56% and 9.42%, respectively. For multi-task recognition, the proposed model outperforms Variant 1 and Variant 2 by 30.50% and 22.14% in accuracy, respectively. This suggests that our proposed MSEI-ENet can recognize complex brain activity patterns even when lower-limb or tongue tasks are involved.

From Figure 11 and Figure 12, it is clearly observed that our proposed model exhibits significant performance improvement across all MI tasks. For the binary left/right-hand MI task, the three models (Variant 1, Variant 2, and the proposed model) perform similarly in the recognition of left-hand and right-hand MI tasks. However, for the multiple classification tasks, there is a noticeable performance disparity among them, which might be because the motor cortex of the lower-limb MI task is located in a deep brain area, which is challenging. This is different from the MI task only involving the upper limbs. Specifically, Variant 1 performs better in recognizing left-hand and both-feet MI tasks, whereas Variant 2 shows better performance in recognizing right-hand and both-feet MI tasks. This indicates that different model architectures have varied recognition abilities for relevant brain activity patterns associated with different MI tasks. Furthermore, Variant 1 and Variant 2 show relatively lower performance on the tongue MI task compared to the other tasks. However, our proposed model significantly improves tongue task recognition, with the metrics of all MI tasks equalling or surpassing 92.66%, and the metric of tongue MI is only 3.09% lower than that of the both-feet MI task. These results demonstrate that the integration of the MSEI and encoder module significantly enhances the effectiveness of MI-EEG decoding. 

(2) Comparison of MSEI-ENet hyperparameters

To compare the influence of different configurations of convolution kernels of the MSEI module on the model performance, Variant 3 and Variant 4 were designed for the ablation experiment. For Variant 3, the convolution kernels of Block 1 of the Main scale were exchanged with Block 1 of the Aux^(1)^ scale, i.e., the kernel sizes of the first three-branch conv2d block of the Main scale were (125, 1), (64, 1), and (32, 1); the sizes of the second one in the Main scale were (32, 1), (16, 1), and (8, 1); and the kernel sizes of the three-branch conv2d block in the Aux^(1)^ scale were (500, 1), (250, 1), and (125, 1). The convolution kernel sizes of the Aux^(2)^ scale were unchanged. 

For Variant 4, the convolution kernels of Block 1 of the Main scale were exchanged with Block 1 of the Aux^(2)^ scale, i.e., the kernel sizes of the first one of the Main scale were (32, 1), (16, 1), and (8, 1); the sizes of the second one were changed to (8, 1), (4, 1), and (2, 1); and the kernel sizes of the Aux^(2)^ scale were (500, 1), (250, 1), and (125, 1). The convolution kernel sizes of the Aux^(1)^ scale were unchanged. 

The related results of the ablation experiment for the left/right-hand MI task on BCIIV 2a are shown in Figure 9. It can be seen that Variant 3 achieves an accuracy of 96.64% and Variant 4 achieves an accuracy of 95.02%, which are 1.62% and 3.24% lower than the proposed model, respectively. From the confusion matrix in Figure 11, the decoding performance of the left and right hands in Variant 3 and Variant 4 have small differences compared with those of the proposed model. We can observe that the variation in the size of the convolutional kernels within the MSEI module has a minor impact on the performance of the model for the left/right-hand MI task.

Figure 10 shows the related results of the ablation experiment for multi-task MI-EEG decoding on BCIIV 2a. Variant 3 achieves an accuracy of 90.65% and a kappa of 0.88. However, the accuracy and kappa values of Variant 4 are 5.91% and 0.07 lower than those of the proposed model. Figure 12 shows the confusion matrix of the multi-task MI-EEG decoding. We can observe that the metrics of the four MI tasks of Variant 3 and Variant 4 are distinctively inferior to those of the proposed model. Among them, the metric of the tongue MI task of Variant 4 declines severely. This indicates that the model is more sensitive to the changes in the convolution kernel size in multi-task MI-EEG decoding. Designing appropriate convolution kernel sizes for different tasks has an influence on the performance of the network. Additionally, regardless of binary or multiple classification tasks, the *PR*, *RE*, and *F*1-score of each class can be ordered from the lowest to the highest as follows: Variant 4, Variant 3, and proposed model. As the convolution kernel size of the Main scale decreases, the performance of the model becomes worse. This indicates that convolutional kernels achieve decent feature learning only when the relevant parameters are appropriately configured.

**Figure 13:**
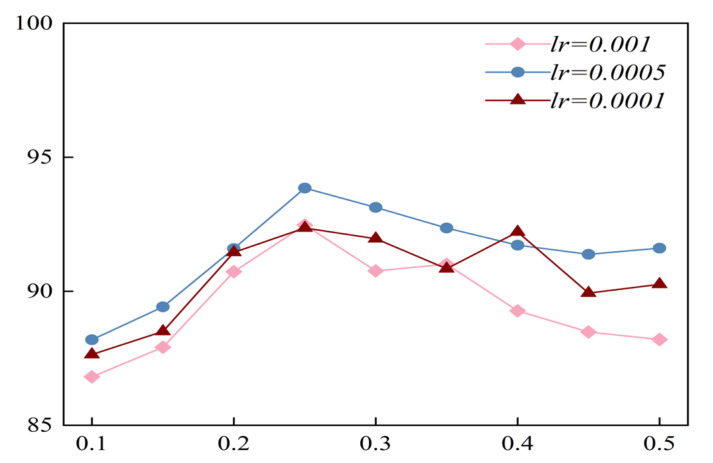
Results of the optimization process of EEG-inception on the test set.

The learning rate (*lr*) and dropout rate are two important hyperparameters in deep learning models. An appropriate learning rate can enable the model to converge quickly to an optimal solution. Moreover, dropout can effectively prevent overfitting in complex networks. Hence, we tested the influence of variations in these two hyperparameters on the performance of the model (*lr* ∈ {0.001, 0.0005, 0.0001}; dropout rate ∈ {0.1:0.05:0.5}). The result of the optimization process on the test set under the scenario of global-CV is shown in Figure 13. We can conclude that under the setting of the same learning rate, the variation in dropout has a distinct influence on the performance of the model (the difference between the maximum and minimum accuracy values is approximately 8%). The reason might be that a too-high dropout rate would cause a substantial number of neurons to be dropped during training, resulting in a deterioration in the learning ability of the model. On the other hand, a too-low dropout rate might cause a degraded ability of the model to predict new data. Additionally, when the dropout rate was unchanged, the accuracy of the model with a learning rate of 0.0005 was generally higher than those with a learning rate of 0.001 and 0.0001. This implies that if the learning rate is too high, it may cause severe instability. Conversely, if the learning rate is too low, learning might become stuck with a high-cost value. 

#### 3.2.3. Comparison of MSEI-ENet with Other Models

To verify the rationality and classification performance of the constructed network, this section compares MSEI-ENet with other models. We conducted experiments by using six state-of-the-art models on the BCIIV 2a and Physionet datasets with the same experimental design. These six models include EEGNet, EEGinception, MMCNN, SMTransformer, Conformer, and Deformer. The first three belong to the CNN framework; the others combine a CNN with Transformer. Table 9 lists the corresponding performance indices of accuracy and kappa using comparison models on multi-task MI-EEG decoding. It can be seen that the proposed model MSEI-ENet achieves a remarkable accuracy of 94.30% and a kappa value of 0.92 on the BCIIV 2a dataset. On the Physionet dataset, MSEI-ENet also achieves good results with an accuracy of 90.48% and a kappa of 0.87. For both datasets, it can be concluded that the performance of the combination models (referred to as CNN with Transformer) is superior to those of CNNs (EEGNet, EEG-inception, and MMCNN). The reason is that the multi-head attention mechanism of Transformer can extract global features and help elevate the performance of the combined models on challenging multi-task MI-EEG decoding. Notably, our proposed model MSEI-ENet outperforms SMTransformer, Conformer, and Deformer, this might be because the employment of a single-branch structure in the CNN limited their efficacy of feature learning. In contrast, the multi-scale structure of MSEI-ENet can extract features more comprehensively, and the adoption of the mechanism of ECA can screen the features of important channels automatically. 

To further validate the performance of our proposed model on new subjects, we performed subject-independent experiments on the BCIIV 2a dataset by using the LOSO-CV method. The related results are shown in Table 10, and the largest value is marked with bold font. It can be seen that by using our model, more than half of the subjects (S1, S2, S3, S6, and S8) achieve a higher accuracy than those of the other baseline models. The accuracy of the proposed model on subject 7 is 2.62% lower than that of EEG-inception. On subject 9, it is only 0.46% lower than that of Deformer, while the accuracies on subjects 4 and 5 are lower than those of the two Transformer models. The possible reason for this phenomenon might be that there is significant individual variability in motor imagery tasks, and the adaptability of the deep learning models to different subjects also varies greatly. However, the average accuracy of our model on the nine subjects is 62.10%, which is higher than that of all the comparison models. These encouraging results demonstrate the effectiveness and robustness of the proposed model in handling the challenges posed by multi-task MI-EEG decoding on new subjects.

## 4. Conclusions

This study proposes a subject-independent MSEI-ENet model for multi-task MI-EEG decoding. The model employs MSEI for original feature extraction, which utilizes three scales to extract spectral and spatial features from the raw multi-channel EEG data. The encoder module further enables discriminative features to be detected by the mechanism of multiple self-attention heads. The ablation experiment indicates that the MSEI module can achieve decent performance compared to the single-scale structure, and the encoder module significantly improves multi-task MI-EEG decoding. These two modules are indispensable for the whole model, and the combination of the two might overcome the coupling effect. The experimental results on the BCI Competition IV 2a show an accuracy of 94.30% and a kappa of 0.92. Additionally, the experimental results on the Physionet dataset achieve an accuracy of 90.48% and a kappa of 0.87, Our proposed MSEI-ENet outperformed the comparison state-of-the-art models. These results show the effectiveness and robustness of the MSEI-ENet model for multi-task MI-EEG decoding. 

## Figures and Tables

**Table 1:** Detailed configuration of the BCIIV2a and Physionet datasets.

Experiments	Datasets		Labels of MI Tasks				
			L	R	F/H	T/F	Total
global-CV	BCIIV2a	Train and Val	8502	8502	8502	8502	34,008
		Test	259	259	259	259	1036
	Physionet	Train and Val	2757	2757	2757	2757	11,028
		Test	84	84	84	84	336
LOSO-CV	BCIIV2a	Train and Val	9445	9445	9445	9445	37,780
		Test	144	144	144	144	576

MI tasks in BCIIV2a involve left hand (L), right hand (R), both feet (F), and tongue (T); MI tasks in Physionet involve left hand (L), right hand (R), both hands (H), and both feet (F).

**Table 2:** Detailed parameters of the Main scale.

Branch	Layer	Filters	Depth	Size	Stride	Padding	Dropout Rate	Input	Output
Branch1	Conv2D block	8	-	(500, 1)	1	same	0.25	*X*	$a_{1}$
	Dw2D block	-	2	(1, 3)	1	-	0.25	$a_{1}$	$b_{1}$
Branch2	Conv2D block	8	-	(250, 1)	1	same	0.25	*X*	$a_{2}$
	Dw2D block	-	2	(1, 3)	1	-	0.25	$a_{2}$	$b_{2}$
Branch3	Conv2D block	8	-	(125, 1)	1	same	0.25	*X*	$a_{3}$
	Dw2D block	-	2	(1, 3)	1	-	0.25	$a_{3}$	$b_{3}$
	Concatenate	-	-	-	-	-	-	$b_{1}$, $b_{2}$, $b_{3}$	*-*
	AvgPool2D	-	-	(4, 1)	-	-	-	*-*	*x*
Branch1	Conv2D block	8	-	(500/4, 1)	1	same	0.25	*x*	$c_{1}$
Branch2	Conv2D block	8	-	(250/4, 1)	1	same	0.25	*x*	$c_{2}$
Branch3	Conv2D block	8	-	(125/4, 1)	1	same	0.25	*x*	$c_{3}$
	Concatenate	-	-	-	-	-	-	$c_{1}$, $c_{2}$, $c_{3}$	*-*
	AvgPool2D	-	-	(2, 1)	-	-	-	*-*	*e* ^1^
	ECA block	-	-	-	-	-	-	*e* ^1^	*E* ^1^

**Table 3:** Detailed parameters of the Aux^(1)^ scale.

Branch	Layer	Filters	Size	Input	Output
Branch1	Conv2D block	4	(125, 1)	*X*	$a_{1}$
	Dw2D block	-	(1, 3)	$a_{1}$	$b_{1}$
Branch2	Conv2D block	4	(64, 1)	*X*	$a_{2}$
	Dw2D block	-	(1, 3)	$a_{2}$	$b_{2}$
Branch3	Conv2D block	4	(32, 1)	*X*	$a_{3}$
	Dw2D block	-	(1, 3)	$a_{3}$	$b_{3}$
	Concatenate	-	-	*b*_1_, $b_{2}$, *b*_3_	-
	AvgPool2D	-	(8, 1)	-	*e* ^2^
	ECA Block	-	-	*e* ^2^	E^2^

The parameters of the Aux^(1)^ scale that are not displayed in Table 3 are consistent with those of the Main scale in Table 2.

**Table 4:** Architectural details of MSEI-ENet.

Layer	Input Size	Output Size	Parameters
Main scale	1000 × 3 × 1	125 × 1 × 24	90,764
Aux^(1)^ scale	1000 × 3 × 1	125 × 1 × 24	1116
Aux^(2)^ scale	1000 × 3 × 1	125 × 1 × 24	456
MSEI module	-	125 × 1 × 72	92,336
Encoder module	72 × 125	72 × 125	345,750
Output module	125 × 1 × 72	4	25,780
Total parameters: 463,866			

**Table 5:** Confusion matrix of binary classification on BCIIV 2a.

	Predicted Labels		Per-Class Metrics		
	L	R	PR	RE	F1
L	254	5	98.45	98.07	98.26
R	4	255	98.08	98.46	98.27

**Table 6:** Confusion matrix of multiple classification on BCIIV 2a.

	Predicted Labels				Per-Class Metrics		
	L	R	F	T	PR	RE	F1
L	242	8	6	3	94.53	93.44	93.98
R	4	247	4	4	92.86	95.37	94.10
F	4	4	248	3	93.94	95.75	94.84
T	6	7	6	240	96.00	92.66	94.30

**Table 7:** Confusion matrix of multiple classification on Physionet.

	Predicted Labels				Per-Class Metrics		
	L	R	H	F	PR	RE	F1
L	75	7	2	0	84.27	89.29	86.71
R	8	74	2	0	84.09	88.10	86.05
H	6	6	72	0	94.74	85.71	89.99
F	0	1	0	83	100	98.81	99.40

**Table 8:** Details of the ablation experiment.

Variant	Model
Variant 1	without the Aux^(1)^ and Aux^(2)^ scales of MSEI and the encoder module
Variant 2	without the encoder module
Variant 3	with the convolution kernels of the Main and Aux^(1)^ exchanged
Variant 4	with the convolution kernels of the Main and Aux^(2)^ exchanged

**Table 9:** Performance comparison of the global-CV experiments for multiple MI classification on BCIIV 2a and Physionet.

Method	BCIIV 2a		Physionet	
	*ACC* (%)	*Kappa*	*ACC* (%)	*Kappa*
EEGNet [15]	57.66	0.44	55.36	0.40
EEG-inception [34]	61.67	0.49	60.83	0.48
MMCNN [21]	80.10	0.73	77.05	0.69
SMTransformer [25]	85.02	0.81	80.92	0.74
Conformer [23]	91.96	0.89	82.85	0.77
Deformer [24]	93.06	0.91	87.62	0.83
MSEI-ENet	94.30	0.92	90.48	0.87

**Table 10:** Performance *ACC* (%) comparison of the LOSO-CV experiments for multiple MI classification on BCIIV 2a.

Method	S1	S2	S3	S4	S5	S6	S7	S8	S9	Average
EEG-inception [34]	60.60	33.35	67.72	44.28	50.36	49.49	**69.98**	55.05	62.51	54.82
MMCNN [21]	68.08	39.78	69.12	50.89	51.06	48.81	54.36	59.05	68.94	56.68
Conformer [23]	58.01	45.34	68.08	53.15	**60.27**	57.67	66.17	60.44	64.61	59.30
Deformer [24]	61.66	46.38	71.38	**54.72**	57.32	52.63	66.87	60.62	**72.25**	60.43
MSEI-ENet	**68.84**	**51.22**	**72.05**	48.44	54.51	**61.81**	67.36	**62.85**	71.79	**62.10**

## Data Availability

The data used are already publicly available.
